# Case Report: Metagenomics Next-Generation Sequencing Can Help Define the Best Therapeutic Strategy for Brain Abscesses Caused by Oral Pathogens

**DOI:** 10.3389/fmed.2021.644130

**Published:** 2021-02-22

**Authors:** Zhonghui Ma, Su Yan, Haoxin Dong, Huifen Wang, Yonggang Luo, Xi Wang

**Affiliations:** ^1^Department of Stomatology, The First Affiliated Hospital of Zhengzhou University, Zhengzhou, China; ^2^Precision Medicine Center, Gene Hospital of Henan Province, The First Affiliated Hospital of Zhengzhou University, Zhengzhou, China; ^3^Health Management Center, The First Affiliated Hospital of Zhengzhou University, Zhengzhou, China; ^4^Department of Infectious Diseases, The First Affiliated Hospital of Zhengzhou University, Zhengzhou, China; ^5^Department of Intensive Care Unit, The First Affiliated Hospital of Zhengzhou University, Zhengzhou, China

**Keywords:** brain abscesses, oral microbiome, gram stain, metagenomics next-generation sequencing, precision treatment

## Abstract

Brain abscesses are associated with an increased long-term risk of new seizures and increased mortality within several years after infection. Common microorganisms that cause brain abscesses include bacteria, fungi, and mycoplasma. We report a 75-year-old man with a brain abscess caused by *Prevotella denticola*, an oral pathogen. Based on the clinical condition, we suspected that the patient had a blood-borne brain abscess, and he received antibiotics and systemic supportive treatment. The patient developed shock for the second time after negative Gram-staining results. Metagenomics next-generation sequencing showed one strain from the oral microbiome, confirming our hypothesis, and targeted antibiotic treatment was administered quickly. Thus, we report a case in which genomic analysis was the critical factor in determining the best antimicrobial therapy for administration.

## Introduction

Brain abscesses are focal infections of the brain, which are associated with an increased long-term risk of new-onset epilepsy and mortality within several years after infection (1). In immunocompetent patients, the microbiome is responsible for most brain abscesses, and the most common pathogenic bacteria are *Streptococcus* and *Staphylococcus* bacteria (2). These bacteria enter the brain either through contiguous spread or hematogenous dissemination, especially in cases of infective endocarditis, or as a consequence of distant infectious foci (e.g., tooth infection) (3, 4).

Changes in the abundance of species disrupt host microbial homeostasis and lead to inflammatory disease (5). Metagenomic next-generation sequencing (mNGS) is used to detect microbial nucleic acids contained in samples via the genomics method, and its most significant advantage is the lack of requirement for culture and premise hypothesis (6, 7).

Herein, we report a case of a brain abscess due to *Prevotella denticola*, an oral periodontitis pathogen half a month after tooth extraction. The patient was diagnosed with intracranial infection based on the clinical indications. The symptoms were significantly relieved after symptomatic treatment for 2 days, followed by sudden and persistent high fever and increased white blood cells. mNGS was used to detect the cerebrospinal fluid samples of the patients and quickly identify the pathogenic microorganisms to administer precision treatment and quickly alleviate the pain in the patient.

## Case Description

We report a 75-year-old man who presented with an unexplained headache persisting for 1 month. Three days prior to admission, the patient presented with dizziness and an aggravated headache, and he did not report any trauma history prior to the onset.

Initially, the patient was admitted to the Department of Neurosurgery. Physical examination showed that his underarm temperature was 38°C, pulse was 105 beats/min, and nervous system showed negative signs. Blood routine examination (BRE) revealed 33,700 leukocytes/μL (91% neutrophils), and brain computed tomography (CT) showed lesions in the left temporal lobe. Hence, he was preliminarily diagnosed with a brain abscess. Treatment with biapenem (0.3 g, twice a day) and vancomycin [1 g, three times a day administered via intravenous (IV) drip] was initiated. One day later, the patient was transferred to the intensive care unit (ICU) as he presented with sudden shock without obvious inducement (low blood pressure, fast heart rate, and abnormal heart rhythm).

On arrival, the patient's body temperature reached 38.1°C and blood pressure was 105/65 mmHg. The patient had poor consciousness and poor spirit, and BRE showed C-reactive protein (CRP) 61.10 mg/L and 9,950 leukocytes/μL (93.7% neutrophils). The cerebrospinal fluid (CSF) was pale yellow and clear, and Gram staining of CSF displayed until 4 days later showed no bacterial growth. Therefore, the treatment regimen included vancomycin (1 g, IV, twice a day), antithrombotic and anti-heart failure agents, and systemic nutritional support. Three days later, his body temperature increased and heart rate was 67 beats/min, while his consciousness was clear, BRE showed a C-reactive protein (CRP) level of 8.35 mg/L, and his leukocyte count returned to normal. However, the patient developed a sudden and persistent high fever on day 6 of ICU arrival. BRE of the patient showed 10,110 leukocytes/μL (16.7% monocytes, 2.9% basophils) and 7,420 neutrophils/μL. Meanwhile, the erythrocyte count decreased to 3,410,000/μL. The CSF presented with a yellow and muddy appearance, with cytology showing 860 leukocytes/μL (25% lymphocytes, 3% monocytes, 72% neutrophils) and a CRP level of 6.1 mg/L.

Meanwhile, mNGS technology was used to detect DNA pathogenic microorganisms in the CSF, and the results showed that the relative abundances of the two oral bacteria were remarkably elevated (Table 1).

**Table 1:** Pathogenic microorganisms isolated in this study.

**Type**	**Genus**		**Species**			
	**Name**	**Sequence number**	**Name**	**Sequence number**	**Relative abundance**	**Attention**
G-	Prevotella	36	*Prevotella denticola*	32	9.80%	High
G-	Fusobacterium	7	*Fusobacterium nucleatum*	3	0.26%	Low

**Figure 1:**
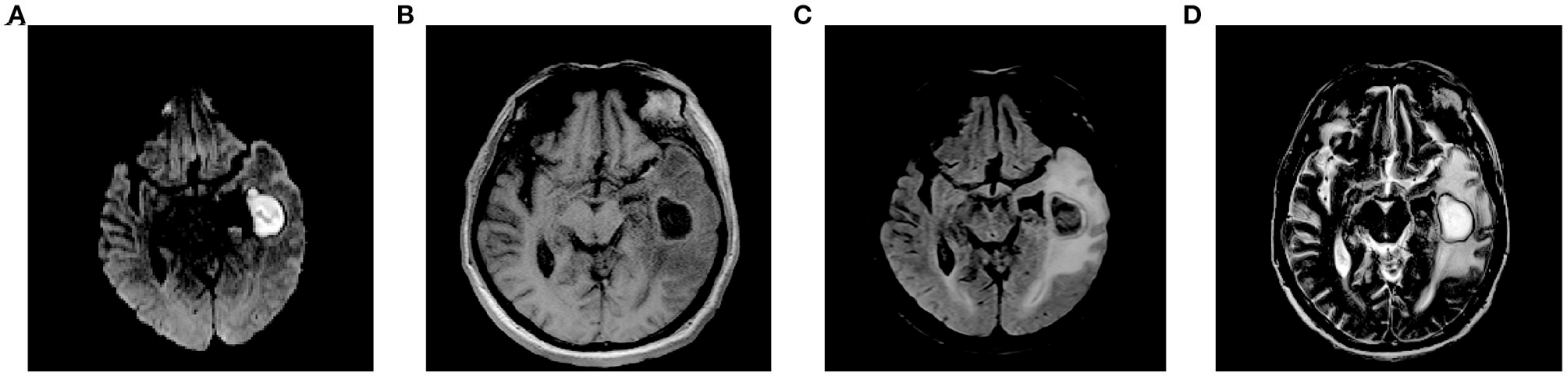
Brain MRI before mNGS. **(A)** DWI; **(B)** T1WI; **(C)** T2-FLAIR; **(D)** T2WI. MRI, magnetic resonance imaging; mNGS, metagenomic next-generation sequencing; DWI, diffusion-weighted imaging; T1WI, T1-weighted imaging; FLAIR, fluid-attenuated inversion recovery; T2WI, T2-weighted imaging.

**Figure 2:**
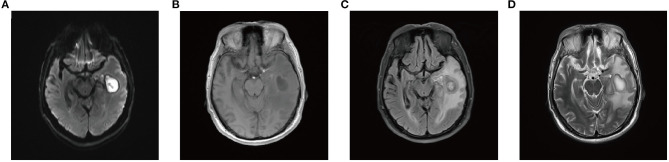
Brain MRI 10 days after mNGS. **(A)** DWI; **(B)** T1WI; **(C)** T2-FLAIR; **(D)** T2WI. MRI, magnetic resonance imaging; mNGS, metagenomic next-generation sequencing; DWI, diffusion-weighted imaging; T1WI, T1-weighted imaging; FLAIR, fluid-attenuated inversion recovery; T2WI, T2-weighted imaging.

After repeated questioning, the patient finally admitted to having undergone a tooth extraction 1 month prior. Brain magnetic resonance imaging (MRI) revealed a left maxillary sinus cyst in addition to the brain abscess (Figure 1). Therefore, the doctor speculated that this was the primary cause of the most recent brain abscess. In general, after tooth extraction, *P. denticola* and *Fusobacterium nucleatum* in the oral cavity enter the blood circulation through the wounds of tooth extraction, return to the intracranial region, and finally cause brain abscesses. After identifying the new pathogenic bacteria, vancomycin was discontinued. The antibiotic treatment was changed to ornidazole (500 mg, IV, twice a day), piperacillin (4.5 g, IV, four times a day), and rifampicin (0.30 g, IV, once a day). One day later, the patient's temperature returned to normal, symptoms improved significantly, BRE showed that the leukocyte count returned to normal, and specialists suggested that the patient could be transferred to an ordinary ward after 2-day infection control. On the 7th day, the patient's brain MRI showed that a sheet low-density shadow in the left temporal lobe of the brain was decreasing (Figure 2). Finally, the patient was followed up to ensure that he did not relapse after 10 days, 1 month, and 3 months. The patient was in good mental condition, had normal physical signs, and did not develop recurrence through January 10, 2021.

## Discussion

Brain abscesses are frequently caused by the continuous spread of concomitant infection areas (e.g., the middle ears, mastoids, and sinuses), and the most common clinical manifestations are headache and fever, which can cause shock in severe cases (2). The most frequent sources of infection in brain abscesses are *Staphylococcus* and *Streptococcus* bacteria. In recent years, some studies have demonstrated other sources of infection, including anaerobes, Gram-negative bacilli, oral *Methanobrevibacter*, associated *Actinobacillus*, bacillus, clostridium, and fungi (8–11).

Currently, CT with contrast enhancement and MRI are the clinical diagnostic methods implemented for brain abscesses. The former provides a rapid means to detect the size, number, and location of abscesses (2). Meanwhile, combined with diffusion-weighted and dispersion coefficient images, MRI is a valuable diagnostic tool for differentiating brain abscesses from primary, cystic, or necrotic tumors (12). At present, abscess puncture, neurosurgery, and antimicrobial agents are the main means of treatment. A stereotaxic navigation system based on volumetric CT or MRI can be applied to the puncture and drainage of abscesses through carefully planned trajectories (13). This system is especially suitable for patients with abscess diameters >1 cm, and abscess puncture is a necessary condition for determining pathogenic bacteria in neurosurgery (3). Neurosurgery is mainly used for superficial abscesses, abscesses that cause brain displacement, or herniation (3).

Antibiotic choice and duration depend on the type of pathogen and severity of infection (8). Brouwer et al. suggested that the selection of initial antimicrobial therapy should be based on the most likely causal organisms of the disease, basis of the mechanisms of infection, patient's predisposing condition, patterns of antimicrobial susceptibility, and ability of the antimicrobial agent to penetrate the abscess (3). In addition, due to the poor sensitivity of current techniques for identifying microorganisms, many experts advocate that antibiotics should cover anaerobes, pending organism identification and the results of *in vitro* susceptibility testing. The combination of third-generation cephalosporin and metronidazole for 6 weeks may treat community-acquired brain abscesses in most immunocompetent patients (14). It is worth mentioning that, besides these two antibiotics, vancomycin should be added to treat potential staphylococcal infection in patients with brain abscesses due to hematogenous spread (8).

Currently, the conventional methods for determining the microorganisms in CSF, blood, or abscesses include aerobic and anaerobic cultures and Gram staining. However, the rapid diagnosis of pathogens in brain abscesses remains a challenge. Experts suggest that the current Gram staining technique is not sufficiently sensitive (14). Al Masalma et al. found that when this test was performed on aspirates from brain abscesses in 71 patients, the positive cultures only corresponded to 30 (42%) (9). Similarly, in this case, negative Gram-staining results prevented the patient from receiving precision antibiotic treatment, leading to the recurrence of the brain abscess. In a previous report, based on the patient's history of present illness, although a bacterial brain abscess was strongly suspected, the culture result was negative, and polymerase chain reaction (PCR)-based 16S ribosomal RNA sequencing provided a definitive etiological diagnosis, allowing for targeted antimicrobial therapy (15). Over the past two decades, the diagnosis rate of pathogenic microorganisms in patients with encephalopyosis has remained poor, while the detection technology (e.g., PCR, antigen assays) of pathogen microorganisms has made remarkable progress (16).

mNGS refers to the interrogation of all the genetic material in an environmental sample. It can be used to identify multiple pathogens (viruses, bacteria, fungi, or parasites) from cultures or directly from clinical samples based on uniquely identifiable DNA and/or RNA sequences (17). Over the past few years, with the increasing maturity and extensive promotion of mNGS technology, the time required to complete initial data analysis has been dramatically reduced from a few weeks to 5–20 min (16, 18). To date, clinical applications of metagenomics have included the diagnosis of infectious diseases for various syndromes and sample types, microbiome analysis in diseases and health control, identification of human host responses to infection through transcriptomics, and identification of tumor-related viruses and their genomic integration sites (6). Thus, RNA and DNA extracted from CSF and brain tissue provide an alternative strategy for the diagnosis of neurological infections via mNGS technology.

In this case, the patient was initially diagnosed with a brain abscess on admission. Meanwhile, based on the patient's physical condition being classified as an emergency, he was initially treated with vancomycin and biapenem as antimicrobial agents. Specially, the bacterial culture results of CSF were negative, and there are two reasons that may be speculated. First, antibiotics might have eliminated *Streptococcus* and *Staphylococcus* as common pathogens; second, we speculate that the limitation of Gram staining technology may have led to the absence of other pathogenic bacteria.

Afterward, the Gram-negative bacteria *P. denticola* and *F. nucleatum* that caused shock were quickly identified through mNGS technology detection, and the patient was saved by antibiotics targeted at the pathogenic bacteria. *Prevotella peridotica* is a Gram-negative bacterium, which is generally considered to be an oral colonization bacterium with conditional pathogenicity that usually causes periodontitis and other diseases. In addition, there have also been cases of headaches after tooth extraction in healthy men, and *Prevotella* was found in blood culture, suggesting that *Prevotella* may represent the etiology of brain abscesses in patients with fever, stroke, and a history of tooth extraction (19). Therefore, we speculated that the Gram-negative result may be attributed to problems with the microbial detection technology. Compared with the success rate of mNGS technology detection, the current Gram staining technology, as a clinical detection technology for microorganisms, has a higher failure rate and is more time-consuming. Finally, the most suitable time for treatment is delayed, which confers many disadvantages to patients, clinical diagnosis, and treatment.

In general, mNGS technology has the advantages of speed and precision, although the current price may be slightly high for some patients. With a significant decrease in the cost of mNGS technology and improvements in data analysis efficiency, mNGS may accelerate the transition from laboratory technology to clinical applications in the future. This suggests that, in addition to authenticating the microorganisms that cause brain abscesses, mNGS may also be applied to the identification of microbes corresponding to other symptoms in the future, such as secondary periprosthetic joint infection and respiratory tract infection caused by unknown microorganisms.

## Data Availability Statement

MNGS was performed at Illumina NextSeq 550 platform in Gene Hospital of Henan Province. Metagenomics whole genome shotgun sequences have been uploaded to EMBL accession: PRJEB41159.

## Ethics Statement

The study was approved by the institutional review board of the First Affiliated Hospital of Zhengzhou University. The ethics approval number is 2019-KY-330. The patients provided written informed consent to participate in this study.

## Author Contributions

MZH, LYG, and WX analyzed and interpreted the patient data. YS, DHX, and WHF performed the experiments. MZH and WX analyzed the genomics data. MZH and LYG wrote the manuscript. All authors have read and approved the final manuscript.

## Conflict of Interest

The authors declare that the research was conducted in the absence of any commercial or financial relationships that could be construed as a potential conflict of interest.
